# Regenerative and molecular therapies for myocardial repair (Review)

**DOI:** 10.3892/mi.2026.326

**Published:** 2026-06-03

**Authors:** Divina Mariya Puthooran, Adlin Tom, Anna Anna, Harini Sundaram, Yousra Anwar, Kirtick Poovendran

**Affiliations:** 1 Faculty of Medicine, Tbilisi State Medical University, Tbilisi 0177, Georgia; 2 Faculty of Medicine, Ivane Javakhishvili Tbilisi State University, Tbilisi 0179, Georgia; 3 Faculty of Medicine, David Tvildiani Medical University, Tbilisi 0159, Georgia

**Keywords:** post-myocardial infarction inflammation, colchicine, canakinumab, cardiovascular outcomes, anti-inflammatory therapy, myocardial remodeling, regenerative therapies, myocardial repair

## Abstract

Despite considerable progress being made in reperfusion, drugs and device-based therapies, myocardial infarction remains a primary cause of heart failure due to irreversible cardiomyocyte loss and maladaptive ventricular remodeling. Conventional surgical and interventional methods cannot regenerate functional myocardium, although they can restore perfusion. Limited endogenous cardiac renewal has driven the development of regenerative, molecular and bioengineering-based therapies aimed at myocardial repair after MI. The present narrative overview summarizes current approaches, including gene and RNA therapeutics, cell-based therapies, extracellular vesicles, engineered cardiac patches and pharmacological strategies. Only modest improvements in left ventricular function were shown in early clinical trials employing mesenchymal stromal cells and bone marrow-derived mononuclear cells; these benefits were mostly attributable to immunomodulatory and paracrine effects rather than true remuscularization. Recent advancements, including cardiac progenitor cells, allogeneic platforms and intraoperative delivery during coronary artery bypass grafting, improved safety but showed mixed outcomes. Induced pluripotent stem cell-derived cardiomyocytes and engineered cardiac patches are a step toward structural myocardial replacement with encouraging preclinical and early human safety data; however, problems with arrhythmogenic risk, immunological rejection, scalability and long-term durability remain unresolved. Parallel developments in gene and RNA therapies, particularly cardiotropic adeno-associated viral vectors, lipid nanoparticle-mediated mRNA delivery and RNA interference, have highlighted the importance of vector design, myocardial targeting and appropriate molecular selection, as evidenced by the inconsistent clinical outcomes of SERCA2a-based gene therapy. Pharmacological management of post-myocardial infarction inflammation, fibrosis, metabolism, and cellular senescence promotes regenerative methods by improving the cardiac milieu and decreasing detrimental remodeling. Both regenerative and molecular therapies have shown encouraging effects on cardiac repair, but successful clinical translation remains a work in progress. With ongoing technological advances and carefully controlled clinical studies, these innovative approaches could ultimately provide effective treatments to regenerate the damaged myocardium and improve outcomes for patients.

## 1. Introduction

Ischemic heart disease (IHD) continues to be the leading cause of morbidity and mortality worldwide, accounting for ~9 million deaths each year (1). As reported by the Global Burden of Disease Study 2021, age-related standardized mortality has declined over the past decades; however, the total burden continues to be on the rise, and IHD contributes to one-third of cases of heart failure (HF) globally (1). Despite prompt revascularization and treatment strategies, acute myocardial infarction (MI) results in marked cardiomyocyte death. The resulting injury triggers a strong inflammatory response, promoting cardiac fibrosis, leading to pathologic ventricular remodeling and progressive HF (2).

The recent study by Derks *et al* (3) demonstrated that cardiomyocyte renewal is markedly reduced in diseased hearts, with renewal rates 18- to 50-fold lower than in a healthy heart. Standard surgical and interventional techniques, including coronary artery bypass grafting (CABG) and left ventricular (LV) restoration, mainly focus on improving blood flow and relieving symptoms; however, they cannot restore the contractile mass of the scarred myocardium. In their study, Hwang *et al* (4) reported an inverse correlation between the transmural extent of myocardial scarring and improvement in cardiac function following CABG. Patients with a low myocardial viability and LV enlargement face a high peri-operative risk and poor long-term outcomes following CABG (5). In their retrospective study, Nakae *et al* (6) found that 40% of patients with ischemic cardiomyopathy did not regain sufficient LV function. This further causes an increased risk of all-cause mortality and worse prognosis (6).

The ability of the heart to recover is limited by the permanent loss of cardiomyocytes. This has turned attention toward regenerative therapies, which do not directly generate new cardiomyocytes, but instead mainly function through paracrine mechanisms, the secretion of factors that promote angiogenesis, modulate inflammation and activate local progenitors, thereby supporting myocardial repair. He *et al* (7) found that exosomes from M1 macrophages carry microRNA (miR)-155, which can suppress cardiomyocyte proliferation by acting on the interleukin (IL)-6R/JAK/STAT3 pathway. This suggests that strategies aimed at reducing M1 macrophage activity or limiting miR-155 release may help to promote heart regeneration (7). Early human trials demonstrated the feasibility of regenerative therapies: Allogeneic Muse cell therapy (CL2020) was well-tolerated with functional improvement in patients with ST-segment elevation myocardial infarction (STEMI), and intracoronary and the intravenous delivery of umbilical cord-derived mesenchymal stem cells (MSCs) improved cardiac function at a 1-year follow-up (8,9).

In addition to cell therapy, molecular strategies such as the adeno-associated virus-mediated delivery of sarcoplasmic/endoplasmic reticulum calcium ATPase 2a (SERCA2a) have been studied. Gene therapy was safely administered to patients with HF with reduced ejection fraction receiving LV-assisted device (LVAD) support in the SERCA-LVAD trial, leading to favorable molecular and cellular alterations, even though full functional recovery was not achieved (10). Autologous CD34^+^ stem cells have demonstrated angiogenic and paracrine effects; a potency assay for good manufacturing practices (GMP)-grade ProtheraCytes identified reliable regenerative indicators that facilitate clinical translation (11). It has also been tested to directly integrate regenerative cells into surgical workflows: At a 1-year follow-up, intramyocardial administration of autologous bone marrow-derived stem cells following CABG was safe and practical, increasing quality of life and reducing angina (12).

The gaps in current treatment strategies highlight the need for regenerative approaches focusing on myocardial repair and regeneration. Although multiple studies have recently entered preclinical and early clinical testing, the evidence is scattered. The present review summarizes current regenerative strategies, examines clinical evidence, and identifies key challenges and future directions.

## 2. Cell and exosome therapies

### Overview and biological rationale

MI is a significant cause of morbidity resulting from ischemic injury and cardiomyocyte necrosis. Stem cell therapy attempts to repair myocardial injuries and restore cardiac function by remuscularization, paracrine signaling, and exosome release. Key cell types explored include bone marrow-derived mononuclear cells (BM-MNCs), MSCs, cardiac progenitor cells (CPCs), induced pluripotent stem cell-derived cardiomyocytes (iPSC-CMs) and skeletal myoblasts (13-16). Extracellular vesicles (EVs) or exosomes, which contain bioactive compounds with immunomodulatory, anti-apoptotic, anti-fibrotic and angiogenic properties, are secreted by stem cells (17-19). Delivery methods aim to optimize myocardial preservation and include intracoronary infusion, intramyocardial injection (epicardial or transendocardial) and intrapericardial administration (20). Preclinical studies consistently support greater angiogenesis, reduced apoptosis and improved cardiac function following MSC-EV, bone marrow cells and iPSC-CM therapy, providing a rationale for clinical translation (14,17,19).

### Clinical evidence: Past and present trials. Early BM-MNC trials

Several trials have investigated the intracoronary infusion of autologous BM-MNCs to improve left ventricular function in patients with MI. REPAIR-AMI demonstrated an ejection fraction (EF) improvement of ~3-4%, with clinical benefits beyond EF changes (16,21). A meta-analysis of 22 randomized controlled trials (RCTs) using BM-MNCs reported the following: LV ejection fraction (LVEF) +2.10% (95% CI, 0.68-3.52), LVESV -4.05 ml, and infarct size-2.5%; magnetic resonance imaging (MRI)-derived outcomes (n=9) showed no improvement. No impact on major adverse cardiac and cerebrovascular events was observed at 6 months. Limitations included heterogeneity, reliance on echocardiography and modest efficacy (21).

The BAMI Phase III trial (n=375, LVEF ≤45%) tested BM-MNCs 2-8 days post-percutaneous coronary intervention (PCI); after 2 years, mortality 3.26% vs. 3.82%, HF hospitalization 2.7% vs. 8.1%. The study was insufficient to detect clear efficacy conclusions (22).

*MSC trials*. MSCs were initially believed to replace damaged cardiomyocytes; it has since been demonstrated that they largely mediate repair through exosome release (14). Previous preclinical studies discussed in the literature have shown that MSC-derived exosomes promote angiogenesis, minimize apoptosis and improve cardiac function (23). A 2021 meta-analysis of 13 RCTs [n=956 patients with acute myocardial infarction (AMI)] found MSCs enhanced LVEF by +3.78% (95% CI, 2.14-5.42), with early treatment (within 1 week post-AMI) providing a benefit of ~5.7% (23). The majority of trials used intracoronary administration; others used intramyocardial or intravenous methods. Heterogeneity in cell doses (2.3-85x10^6^), infusion timing and follow-up (6-182.6 months) limited comparisons. No significant effects were observed on LV volumes or HF rehospitalization (23). Preclinical evidence suggests that MSCs also activate endogenous cardiac stem cells, promoting proliferation, migration, differentiation, niche reconstruction and remodeling of necrotized tissue (24).

Trials, such as POSEIDON and TAC-HFT evaluated allogeneic and autologous MSCs in chronic ischemic cardiomyopathy and revealed scar reduction, safety and functional improvements, particularly following a transendocardial injection. MSC-HF demonstrated improved symptoms, but no notable EF effect. Overall, the benefits were moderate and were influenced by delivery-related variability (25).

*Trials for CPCs*. Trials have demonstrated that CPCs such as c-kit^+^ and cardiosphere-derived cells (CDCs) promote myocardial regeneration. The stem cell infusion in patients with ischemic cardiomyopathy (SCIPIO) trial reported improved LVEF (from 30% to ~38%) and a reduced infarct size following the intracoronary injection of autologous c-kit^+^ CPCs in ischemic cardiomyopathy (26). The cardiosphere-derived autologous stem cells to reverse ventricular dysfunction (CADUCEUS) trial demonstrated very minor EF alterations using CDCs, but a significant scar reduction (-12 g) with corresponding increases in the viable myocardium (27). ALCADIA, which paired CDCs with a gelatin hydrogel, revealed improved EF and symptomatic improvement despite the limited sample size (28). The current evidence remains mostly preclinical compared to BM-MNC and MSC studies, revealing a huge knowledge gap (21).

*First-in-human intraoperative trials*. Intraoperative trials are used to evaluate direct, targeted cell administration during cardiac surgery. PROMETHEUS, a feasibility study that injected autologous MSCs during CABG, revealed enhanced regional wall motion and procedural safety but was not powered for efficacy (29). CONCERT-HF, testing CDCs and MSCs, demonstrated improvements in quality-of-life outcomes and a reduction in HF hospitalization, but only marginal improvements in ventricular function (30). DREAM-HF, a large phase III study, demonstrated reductions in inflammatory biomarkers and fewer hospitalizations for heart failure, particularly in patients with high inflammatory status, but no improvement in the primary composite outcome (31).

Autologous bone marrow-derived MSCs administered via CABG were used in transmyocardial revascularization + bone marrow-derived MSC trials. Patients under FDA BB-IND 14758 exhibited feasible and short-term safety (12).

*Meta-analyses and translational lessons*. Pooled BM-MNC and MSC studies have revealed modest LVEF improvements (~2-4%), with LV volumes, infarct size, and medical results (mortality, reinfarction and HF hospitalization) typically unchanged. Observed improvements likely reflect paracrine effects rather than remuscularization, underscoring the reason for cell-free therapies (e.g., exosomes) or better delivery systems (21,23,32). Key clinical trials testing regenerative therapies are summarized in Table I.

### iPSC-derived cardiomyocytes: Preclinical to early clinical

iPSC-CMs are promising for cardiac remuscularization as they can electrically integrate with host myocardium and form force-producing grafts capable of enhancing ventricular function. Preclinical large-animal studies, particularly in non-human primates, have demonstrated an enhanced LVEF and the remuscularization of the infarcted myocardium (33,34). However, transient ventricular arrhythmias can be caused by immature electrophysiology and insufficient host-graft coupling. Immunologic rejection is more likely in allogeneic transplants without immunosuppression or hypoimmunogenic editing (33,34). Some strategies to reduce risks include promoting iPSC-CM maturation, improving gap-junction formation (e.g., connexin-43), producing conduction-synchronized sheets, selective immunosuppression and generating HLA-edited universal donor lines (33,34). First-in-human iPSC-CM patch implantation has demonstrated early viability and safety (35,36). The remaining concerns are immunological tolerance, scalable production, long-term graft durability and arrhythmogenic risk (37).

### Exosomes/EV approaches

EVs rich in lipids, proteins and nucleic acids are essential for the regulation of myocardial injury and repair. Stem cell-derived exosomes possess the same angiogenic, anti-fibrotic, anti-apoptotic and immune-modulating characteristics as parent cells (17). Preclinical MSC-EV research in rodent MI models has revealed increased angiogenesis, reduced apoptosis and improved LV function (14). Therapeutic translation is limited by low tissue retention, unstable biological activity, low manufacturing yield and insufficient homing efficiency (19). To overcome these limitations, delivery innovations include cardiac patches, hydrogels loaded with exosomes, and microneedle devices to enhance retention and myocardial regeneration. Engineered exosomes with increased tropism or therapeutic cargo further enhance targeting (38,39). Clinical studies evaluating EV treatments, such as cardiosphere-derived vesicles (CAP-1002) in Duchenne muscular dystrophy, demonstrate safety and early translational potential despite the lack of MI-specific trials (40). While the risk of arrhythmia is lower than with direct cell therapy, safety monitoring is still critical (41).

## 3. Tissue-engineered cardiac patches and biomaterials

**Figure 1:**
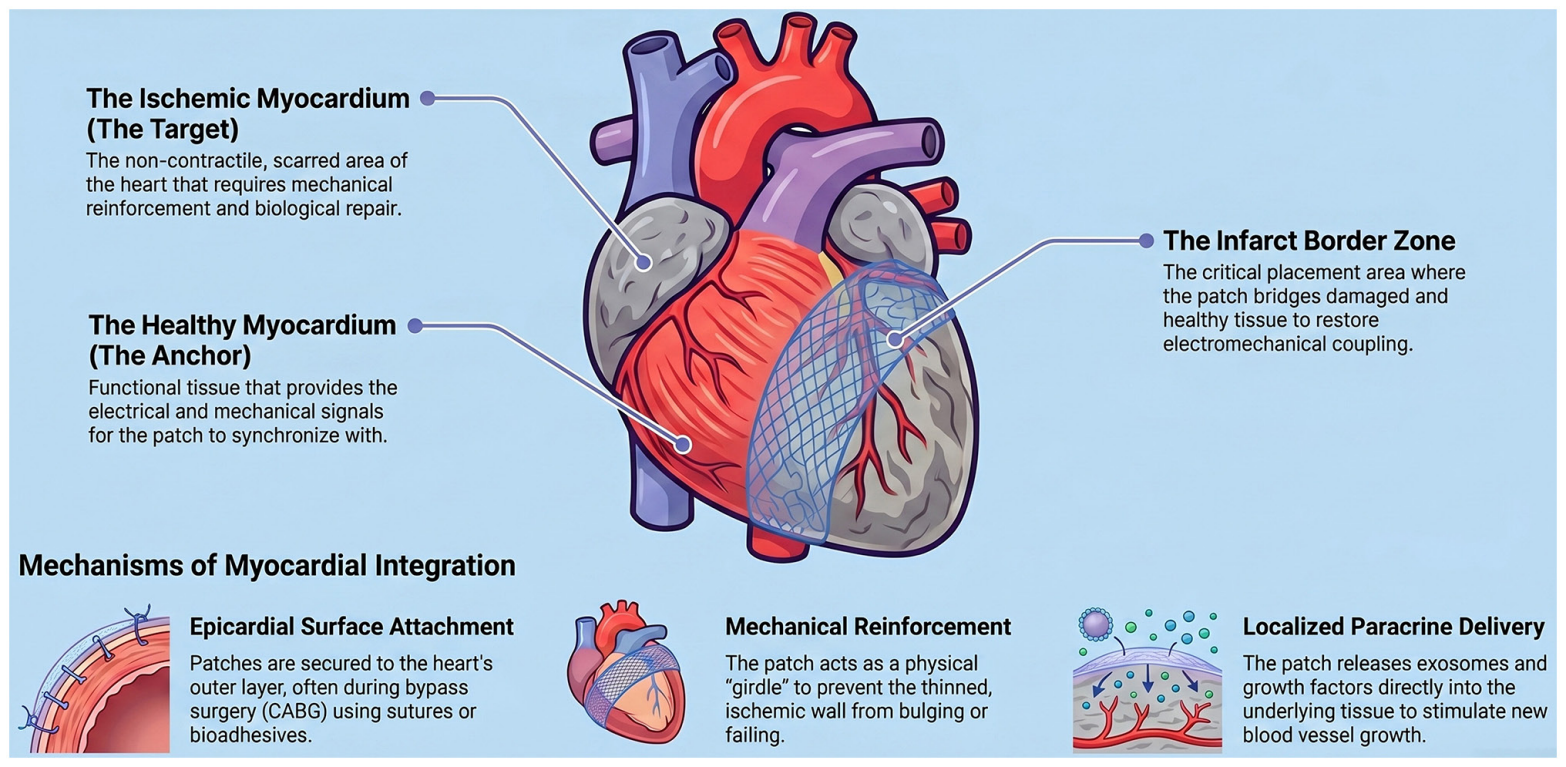
Schematic representation of engineered cardiac patch placement and mechanisms of myocardial integration following ischemic injury. Cardiac patches are positioned over the infarct border zone to bridge ischemic and healthy myocardium, providing mechanical reinforcement, electromechanical coupling and localized paracrine signaling.

Cardiac tissue engineering aims to restore myocardial form and function by directly introducing biomechanically and physiologically active structures to injured myocardium (41-43). Unlike systemic cell delivery, which is limited by poor retention and survival, epicardial cardiac patches are designed to provide better electromechanical integration at the infarct border zone, persistent paracrine signaling, and localized mechanical reinforcement (44,45). Engineered cardiac tissues derived from human induced pluripotent stem cells (hiPSCs) enable contractile remuscularization, disease modeling and pharmacological testing; however, scalability and safety difficulties continue to restrict their clinical use (46). The positioning of engineered cardiac patches relative to the ischemic and healthy myocardium is illustrated in Fig. 1.

### Types and design goals

Tissue-engineered cardiac patches can be broadly classified into four functional classes: Injectable hydrogels, decellularized extracellular matrix (ECM)-based epicardial patches, electroconductive scaffolds and three-dimensional vascularized constructs. Injectable hydrogels provide minimally invasive distribution, often via the pericardial space, and primarily serve as temporary bioactive depots for cells or growth hormones rather than load-bearing structures (47,48). Decellularized extracellular matrix-based epicardial patches preserve native nanoscale architecture and biochemical signals, promoting host cell infiltration and angiogenesis while avoiding undesirable ventricular remodeling (49). Electroconductive patches employ materials, such as graphene derivatives or conductive polymers to increase electrical signal conduction across infarcted myocardium and address conduction block and desynchrony that limit functional recovery (50,51). Engineered three-dimensional (3D) vascularized cardiac tissues, which aim for partial myocardial replacement, constitute the most ambitious strategy. However, difficulties with thickness scalability, rapid perfusion and surgical integration limit their translation (52).

Biocompatibility, regulated biodegradation, mechanical compliance that mimics natural myocardium, sufficient tensile strength for enduring cyclic loading, and, when required, electrical conductivity are common design goals for all patch categories. These traits influence cell survival, host integration, and long-term functional value (51,53-55). Electroconductive scaffolds with high biocompatibility also aim to reduce the need for sutures and staples, which increase the risk of bleeding, trauma and infection (56).

### Preclinical evidence and fabrication methods

Common fabrication methods include molding, electrospinning, 3D printing, spray drying and *in situ* crosslinking. Nanofibrous polyester or biopolymer-based patches with fiber alignment that resembles natural epicardial collagen are frequently made via electrospinning, giving cells structural cues (43). Complex patch structures utilizing biomaterial inks, such as thick cardiac constructions with anisotropic fibers and perfusable vascular channels, are now attainable owing to advancements in 3D printing (43,57). To endure constant epicardial motion, cardiac patches must maintain their toughness, flexibility, and fatigue resistance regardless of the manufacturing process (43).

Patch biomaterials range from natural polymers, such as fibrin, gelatin, alginate, chitosan, collagen, hyaluronic acids, silk and decellularized ECM to synthetic polymers, such as polyglycolic acid, polylactic acid, polylactic-co-glycolic acid, polyurethane and their derivatives (43,58). While synthetic materials offer customizable mechanics, consistent production, simpler processing and less immunogenicity, natural materials have marked biocompatibility and promote cell attachment and proliferation (43).

Recent preclinical studies have demonstrated that the *in vivo* efficacy of cardiac patches is directly impacted by fabrication decisions. Melt electrowriting and volumetric 3D printing yielded a reinforced patch with high mechanical strength, enabled by a poly (ε-caprolactone) metamaterial and cell infiltration assisted by the infiltrated fibrin hydrogel. The implant demonstrated suturable implantation feasibility and acute functional support in a large-animal model of acute MI by withstanding intraventricular pressure, lowering peak ventricular pressure and preventing bleeding (59). Aligned electrospun rGO/PLCL membranes confirmed the possibility of using conduction-consistent cardiac patches to improve drug screening and disease modeling applications (60). Apart from enhancing the electroactivity of the infarcted heart, a self-powered biomimetic trinity triboelectric nanogenerator conductive cardiac patch can wirelessly monitor electrocardiosignals (61).

### First-in-human and early clinical implementations

First-in-human and early clinical studies are currently beginning to translate epicardial cardiac patches from the laboratory to clinical practice. The first-in-human trial with cardiac bio-patch implantation was PeriCord, which comprised a scaffold based on decellularized pericardial tissue and Wharton's jelly MSCs (WJ-MSCs). The bio-patch was secured onto the epicardium through surgical glue during simultaneous CABG surgery, demonstrating that the patch can be integrated easily into standard open-heart workflows. The implantation was described as ‘uneventful’ with no severe adverse reaction, no arrhythmias, no signs of pericarditis and no need for any immunosuppression. Through migration, transdifferentiation and paracrine actions, WJ-MSCs promote revascularization, decrease infarct size, alleviate unfavorable remodeling and fibrosis progression, and enhance cardiac function. Three consecutive batches of PeriCord were manufactured, all of which satisfied the acceptance criteria for cell dosage and viability, and the study shows good manufacturing practices. As a preliminary, single human implantation, it lacks long-term efficacy data and a control group comparison. The beneficial effect of this bioimplant will be validated by a long-term follow-up and the outcome of the PERISCOPE study (62).

The Xeltis pulmonary valve (XPV) is a biorestorative, bioabsorbable valved conduit evaluated as a pediatric pulmonary valve replacement. Following early concerns with leaflet prolapse and regurgitation in the first 12 XPV-1 implants, a revised XPV-2 was implanted in 6 children. Growth factors and cell seeding were not necessary for the commercial conduits (16-18 mm). At 12 months, all 17 of the 18 patients were released within 7-10 days and exhibited no signs of stenosis, dilatation, aneurysm, or early adverse effects. Histology verified that undamaged, non-inflammatory leaflets were consistent with in situ tissue repair. XPV-2 also exhibited reduced pulmonary insufficiency and considerably increased fatigue life in bench tests, supporting extended functional recovery. Limitations include small sample size, multicenter heterogeneity and short follow-up, which prevent verdicts about long-term durability or growth potential. Although the XPV is not a myocardial patch, it is an excellent example of a strong translational example of this type of ‘biorestorative’ vascular conduit to enter human clinical practice (63).

Bhatt *et al* (64) described the usage of a second-generation CorMatrix ECM patch that was implanted in a patient with a large anterior MI. After median sternotomy for CABG, the patch was stitched onto the epicardial membrane. The following day, satisfactory short-term recovery led to extubation. Post-operative imaging by late gadolinium enhancement (LGE) cardiac MRI revealed progressive remodeling and an initial subendocardial scar, with LVEF increasing from 10% pre-operative to 51% at 14 months and a noticeable reduction in LGE extent. The single concern was unrelated diffuse anoxic brain damage, but no patch-related safety problems, such as infection, arrhythmia, or patch failure, were reported (64). ECM patches may function through mechanical constraints to limit remodeling and distribution of paracrine substances (growth factors and matrix-bound vesicles), promoting angiogenesis, resident cell mobility, and reducing inflammation/scarring. According to Bhatt *et al* (64), further clinical observational studies or large-scale clinical trials are necessary to validate or refute the effects based on a single patient case report. Although first-in-human experiences reveal procedural feasibility and encouraging early safety, data for therapeutic efficacy remain anecdotal and extremely preliminary, reflecting the variable maturation and translational readiness of epicardial biomaterial therapies.

### Delivery techniques and intraoperative workflow. Open approaches

The most popular technique for delivering epicardial patches continues to be open surgical insertion. Goldman *et al* (65) proposed to implant the tissue-engineered patch on the epicardial surface of the heart in patients undergoing elective on-pump CABG via median sternotomy who have poor LV function. The selection of this CABG patient population allows for patch implantation with minimal additional surgical risk, since the epicardium will already be exposed during the CABG procedure (65). Currently, patches are mostly transplanted during procedures such as CABG that require open access to the chest. However, developments in adhesion design and shape-memory materials may open the door to minimally invasive patch implantation via transapical puncture, thoracoscopy, or even percutaneous coronary intervention (43).

*Minimally invasive robotic approaches and device-assisted delivery*. The HeartStamp is a robotic instrument designed for a minimally invasive approach through a uniportal video-assisted thoracoscopic surgery (VATS) approach. Patch application was attempted using two access routes: A postero-inferolateral approach mimicking a typical uniportal VATS and an anterolateral method mimicking a minimally invasive transapical route. The pericardium was closed over the implant once the patch had been positioned and verified to be in place (66).

VATS provides advantages, such as less pain, a shorter hospitalization period and a lower risk of developing post-operative chest infections (67). By contrast, open surgeries such as sternotomy or thoracotomy are associated with prolonged recovery times and higher complication rates (66).

*Surgical relevance*. Delivery methods include direct epicardial suturing during open-heart surgery, bioadhesive fixation using fibrin glues and minimally invasive intrapericardial injection of thermosensitive hydrogels (47,68,69). Bioresorbable anchoring and suture-free systems are being developed to reduce procedural trauma. GelMA/Bio-ionic liquid patches, for instance, create ionic connections with the myocardium, reducing the need for sutures by forming electrostatic interactions between charged functional groups in the hydrogel and oppositely charged components of the epicardial surface, which enhances immediate tissue adhesion and electrical coupling while avoiding mechanical suturing (56). Timing and surgical integration are crucial, as Jabbour *et al* (70) demonstrate rapid vascularization by week 1 following patch implantation. Patches are usually applied intraoperatively prior to the chest closure, and peri-operative care concentrates on hemostasis, placement of a pericardial chest tube and securing ECM patches. Post-operative surveillance focuses on bleeding, effusions, atrial fibrillation and 30-day readmission risks (71).

*Peri-operative considerations*. The advantages of synthetic polymer patches include rapid availability and convenient storage, although they also entail higher risks of thrombogenicity, infection, bleeding and aneurysms (72). Dense adhesions complicate reoperative patients by raising the risk of intraoperative hemorrhage, cardiac or vascular injury and longer operating times with poor surgical outcomes. Emerging hydrogel strategies could help reduce adhesion formation and associated reoperative risks (73).

*Next-generation adhesive technologies*. Prevascularized patches have improved vascularization and muscularization, less fibrosis and more M2 macrophage infiltration than acellular patches, all of which enhance post-repair cardiac function (74). A self-adhesive conductive patch using graphene functionalized with methoxytriethylene-glycol enhances cardiac function after infarction, while preserving minimal immunogenicity (75). The two-layer 3D-printed MagPatch is another breakthrough that facilitates targeted drug administration and rapid vascular repair (76).

*Outcomes and durability*. Preclinical models typically demonstrate reduced fibrosis, enhanced angiogenesis and moderate functional recovery following patch implantation, particularly when the constructions are electrically conductive or prevascularized (77,78). The biological plausibility of modified cardiac muscle transplants is supported by their long-lasting retention and dose-dependent effects without tumorigenicity in large-animal and early human trials (79). However, long-term durability, electromechanical stability, arrhythmogenic risk and scalable manufacturing remain concerns (65).

*Cardiac MRI (cMRI; LGE) and structural outcomes*. The gold standard for evaluating the structure and function of the heart remains cMRI. Nitric oxide-treated hearts revealed smaller infarcts than controls on LGE-cMRI (80). cMRI was used to quantify LV volume, mass, and scar size from baseline to seven months following MI. As infarcted tissue frequently spreads beyond what the surgeon can perceive intraoperatively, correct TE patch implantation is guided by scar localization on one-month imaging (65). Continued long-term cMRI is critical, as cardiac remodeling and patch-host integration can progress beyond six months and affect persistent therapeutic efficacy (68).

*Speckle-tracking echocardiography (STE) and its functional outcomes*. In preclinical cardiac research, STE is a helpful method used to improve data quality and translational relevance by measuring myocardial deformation using post-processed pictures (81).

## 4. Gene and RNA therapies

### Rationale and delivery strategies

Gene and RNA-based therapy aim to modulate angiogenesis, cytoprotection and electromechanical stability via transient, yet durable expression (82). The use if adeno-associated vector (AAV) has proven to sustain expression for longer periods of time, while mRNA delivery via ionizable lipid nanoparticles (LNPs) has streamlined efficiency and lower cytotoxic effects. Systematically delivered LNP-mRNA accumulates in the injured myocardium due to injury-induced endothelial activation (83). In order to optimize myocardial uptake and spatial distribution, there are several delivery routes. Intracoronary infusion delivers therapeutics through the coronary vasculature, thereby covering a larger territory (84). Transendocardial injection amplifies myocardial uptake, exhibits improved retention and subsequent tissue repair post-MI (85). The clinical trial by Anttila *et al* (86) (n=11) highlighted that direct intramyocardial injections of vascular endothelial growth factor-mRNA during CABG procedures enhanced perfusion, decreased fibrosis and improved capillary density. Epicardial gene-eluting patches and bioengineered scaffolds provide sustained and localized release of gene or mRNA vectors during invasive or minimally invasive procedures (43). Collectively, these approaches facilitate interventional teams and surgeons to match delivery modalities to anatomical access and tissue objectives.

### Clinical lessons: AAV/SERCA2a and emerging cardiotropic vectors

The translational trajectory of AAV1-SERCA2a serves as a cautionary clinical example, demonstrating encouraging initial results and yet revealing significant limitations in larger trials. In the study by Jaski *et al* (87) in 2009, the first in human CUPID phase 1/2 study, which included a total of 9 patients with advanced systolic HF, the patients were administered escalating intracoronary doses. Their study demonstrated that AAV1-SERCA2a infusion provided an acceptable safety profile and investigative breakthrough in functional classification, N-terminal pro-B-type natriuretic peptide (NT-proBNP), and left ventricular end-systolic volume, suggesting early biological activity (87). An extended follow-up, a randomized, double-blind, placebo-controlled trial with patients who were treated similarly, revealed that safety and symptomatic and biomarker benefits were maintained throughout 6-12 months. These findings prompted the launch of a larger confirmatory randomized trial (88). The CUPID-2 Phase 2b trial randomized 250 patients (123 AAV1-SERCA2a; 127 placebo); however, the trial yielded clinical neutral results with no reduction in HF-related events, no benefit in composite time-to-event outcomes, and no quantifiable functional advantage despite overall safety. The failure to replicate earlier results highlighted structural limitations of first-generation vectors (89).

Structural analyses point to several factors contributing to this translational gap. First-generation AAV1 exhibits limited cardiotropism, dose ceilings inherent to intracoronary infusion and a reduced number of empty capsids, which otherwise function as immune decoys-causing increased exposure to neutralizing antibodies and impairing gene transmission. Improved SERCA2a upregulation mandates superior delivery systems, tailoring capsid composition and vectors resistant to innate immunity. Additionally, more in-depth analyses reveal that although SERCA2a downregulation is characteristic in heart failure with reduced EF, its role in HF with preserved EF (HFpEF) remains uncertain, where titin-dependent stiffness, metabolic deficits and microvascular inflammation drive core pathophysiology (90).

Novel research currently addresses these gaps. Sasaki *et al* (91) reported that commonly used clinical AAV capsids (for example, AAV1) exhibited low gene-delivery efficiency in human cardiac tissue; in this context, ‘transduction’ refers to successful delivery and the expression of a therapeutic transgene in cardiomyocytes. They further demonstrated that engineered AAV9-based MyoAAV variants (4A and 4E) exhibited improved cardiomyocyte transduction and reduced off-target uptake. This distinction is important because vector tropism and transduction efficiency determine both efficacy and safety of cardiac gene therapy (91). In parallel, Henry *et al* (92) highlighted that the systemic delivery of AAV2i8-based AB-1002 in 11 patients with HF overcame earlier dosing limitations and exhibited improved myocardial uptake. Patients exhibited an increased LVEF (+4 to +7% at 6 months), decreased NT-proBNP and an improved 6-min walk test (avg +28-40 m) (92). These findings collectively suggest that successful cardiac gene therapy requires cardiotropic vectors, greater myocardial exposure and validated molecular targets.

### RNA therapeutics, antisense and gene editing

RNA-based therapeutics have evolved as versatile tools to combat cardiac injury, inflammation and subsequent remodeling. Small interfering RNA (siRNA) and antisense oligonucleotides (ASOs) facilitate accurate post-transcriptional silencing of pathogen targets. In murine models, small EVs engineered to display a cardiac-targeting peptide improved the myocardial uptake of siRNA directed against the receptor for advanced glycation end products (RAGE), thereby reducing inflammatory infiltrates and fibrosis (93). Similarly, optimized siRNA targeting the long non-coding RNA myocardial infarction-associated transcript (MIAT) decreased cardiomyocyte apoptosis, improved post-MI functional recovery and upregulated cardioprotective factors (94). A novel method to improve N-acetylgalactosamine conjugation enhances siRNA potency and stability, supporting translational potential for systemic delivery and, with appropriate targeting, cardiac applications (95).

Beyond siRNA, ASOs have exhibited immunomodulatory efficacy. Macrophage-targeted ASOs against nucleophosmin 1 reprogram macrophage metabolism toward a reparative phenotype, increasing efferocytosis, pro-angiogenic signaling, and extracellular matrix remodeling, which enhances the macrophages' capacity to support myocardial repair after infarction (96). This mechanistic link between macrophage metabolic reprogramming, efferocytosis, and improved infarct healing is supported by recent immunometabolism and efferocytosis reviews (97,98). RNA drugs already used in cardiometabolic disease (for example, inclisiran and volanesorsen) illustrate the clinical maturity of some RNA modalities and help de-risk cardiac translation (99).

mRNA platforms enable transient expression of reparative proteins; modified mRNA encoding vasculogenic or cytoprotective factors has stimulated neovascularization and cardiomyocyte survival in preclinical models (100). miRNA modulation remains complementary: The delivery of miR-93 activates the Hippo-YAP pathway, increasing angiogenesis and reducing infarct size and fibrosis in animal studies (101).

Gene editing is at an earlier translational stage than many RNA therapeutics and viral gene-delivery approaches; the majority of cardiac gene-editing strategies remain preclinical, although allele-specific RNA nuclease approaches, such as clustered regularly interspaced short palindromic repeats (CRISPR)-Cas13d have restored contractility in patient-derived cardiomyocytes, demonstrating proof of concept (102,103). Early CRISPR-Cas9 application in cardiac cells has demonstrated non-specific uptake (104). Due to their reliable pharmacokinetics, ability for scalable production, and the lack of long-term genomic integration, RNA platforms are more advanced therapeutically than viral gene therapy. Major gene and RNA therapy vectors, targets, supporting evidence and limitations are listed in Table II.

### Safety, manufacturing and regulatory perspectives

Safe viral-vector use requires synchronized biosafety supervision, standard personal protective equipment practices, controlled access, vector shedding surveillance and occupational exposure mitigation, which is supported by institutional biosafety committee review (105). Clinical workflows decrease staff exposure and ensure safe handling of biologics, although vector shedding and unintended antibody development remain a concern (106). Regulatory bodies such as the Food and Drug Administration (FDA) focus on vector shedding and environmental monitoring and risk-benefit evaluation (107). Immunogenicity also poses a practical barrier, as the majority of the population carries preexisting neutralizing antibodies to AAVs, which in turn diminishes its therapeutic efficacy. Corticosteroid-based immunosuppression, when administered with or without adjuncts, shows uniform results in reducing immune activation against capsids (108). Manufacturing mandates GMP-compliant vector production along with early interaction with regulatory bodies, and close coordination with clinical teams, quality units to achieve sterility, potency and timely batch release (109). Effective implementation necessitates structured planning between surgical teams, GMP manufacturers, and regulatory bodies to assure safe and regulated distribution. A comparative overview of the major regenerative and molecular therapeutic approaches for myocardial repair, including their mechanisms, efficacy, safety considerations and translational status, is summarized in Table III.

## 5. Novel pharmacological and biological agents that promote myocardial repair

### Anti-inflammatory strategies following MI

During MI, the heart experiences a rapid and intense inflammatory response. This acute inflammatory surge is linked to the pathogenesis of post-MI remodeling. The activation of the NOD-, LRR- and pyrin domain-containing protein 3 (NLRP3) inflammasome during this phase promotes cardiomyocyte injury and fibrotic remodeling, thus increasing the risk of HF in patients post-MI (110).

Colchicine, an orally administered anti-inflammatory medication, functions by inhibiting tubulin polymerization, thereby reducing inflammasome activation and proinflammatory release (110). In the COLCOT trial, patients were randomly assigned to colchicine or a placebo. At a median follow-up of 22.7 months, colchicine significantly reduced a composite of serious cardiovascular outcomes, including MI, stroke, and cardiovascular death by 23% compared to placebo (110).

Similarly, results from the CANTOS trial revealed that canakinumab, an IL-1β inhibitor, lowered the risk for a major cardiovascular event in patients recovering from MI with persistent inflammation. A 20% reduction was observed in the rate of the total number of serious cardiovascular events (111). Both COLCOT and CANTOS were designed around the same principle of targeting the IL-1β/NLRP3 inflammatory axis to reduce maladaptive remodeling.

However, it is important to note that neither trial demonstrated true myocardial regeneration; their benefits were limited to systemic anti-inflammatory effects and reduction of recurrent ischemic events (110,111).

### Anti-fibrotics, metabolic and mitochondrial agents

Cardiac fibroblasts are central to the remodeling process. They differentiate into myofibroblasts and release vast quantities of extracellular matrix proteins, resulting in cardiac fibrosis (112). The majority of cardiac fibroblasts originate from resident fibroblasts, which are derived from epicardium-derived progenitor cells through epithelial to mesenchymal transition, during embryonic development, a process regulated by the myocardin-related transcription factor and serum response factor axis (112). Anti-fibrotic therapy represents another important approach to limiting adverse remodeling. In the PIROUETTE study by Lewis *et al* (113), 94 patients with HFpEF were randomized to receive pirfenidone or placebo. Over a period of 52 weeks of pirfenidone treatment reduced fibrosis and NT-proBNP. The reduction in myocardial extracellular volume fraction was associated with a 9 to 28% decline in hospitalization (113).

Pirfenidone is the first antifibrotic that has been tested in patients with HFpEF. It is orally administered, has minimal side-effects, and functions by suppressing transforming growth factor β (TGF-β) and various pro-inflammatory cytokines [tumor necrosis factor α (TNF-α), IL-4 and IL-13]. However, no changes were observed in growth differentiation factor (GDF)-15 levels. This cytokine is expressed in low levels in normal human tissues. In the heart, cardiomyocytes increase GDF-15 levels in response to oxidative stress and ischemic injury (114).

There has been increasing attention focusing on sodium-glucose cotransporter 2 (SGLT2) inhibitors for their protective mechanisms on the heart. The cardioprotective effects of SGLT2 inhibitors arise from their influence on metabolism, limiting inflammation and affecting myocardial signaling pathways through inhibition of Na^+^/H^+^ exchanger (115). Evidence from clinical trials indicates its safety and efficacy in patients with HF. In the EMMY trial, 476 patients post-MI were randomized within 72 h of PCI to receive empagliflozin at 10 mg daily. Over a period of 26 weeks, empagliflozin led to a greater decrease in NT-proBNP, an increase in LVEF, and improved diastolic function compared with the placebo (115).

Although favorable, the EMMY trial was not designed to assess regeneration, but rather early post-MI biomarker changes (115). Another study reported that SGLT2 inhibitors exhibited better structural heart function. Older adults, men and patients with STEMI had a 42-46% reduction in left ventricular remodeling risk (116).

Mitochondrial-targeted therapies have also shown promise. An *ex vivo* study investigated the effects of elamipretide on human cardiac mitochondria. It showed that elamipretide improved mitochondrial function in failing hearts and enhanced the CI-CIII-CIV supercomplex activity (117). Trimetazidine may improve both functional and mortality outcomes in patients with heart failure. These benefits arise from its ability to inhibit mitochondrial long-chain 3-ketoacyl-CoA thiolase and enhance glucose oxidation (118). This metabolic shift protects myocardial cells from necrotic and apoptotic death by reducing oxidative damage and stabilizing intracellular ion homeostasis (119). In another retrospective study, therapy with trimetazidine was associated with a slight increase in LVEF at 6 months and a reduction in LV filling pressure. The trimetazidine group also had a 70% lower risk of hospitalization (119).

### Senolytics and cellular rejuvenation

With advancing age, cardiovascular tissue experiences increasing senescence due to mitochondrial dysfunction, oxidative stress from reactive oxygen species, DNA damage and telomere deterioration (120). Senescent cells are known to avoid apoptosis by activating multiple pro-survival mechanisms (121). These cells secrete harmful pro-inflammatory and pro-fibrotic signaling molecules collectively, known as senescence-associated secretory phenotype, which hinders regeneration (122). Experimental models of natural ageing have shown that senescence is a critical factor in the progression of age-related cardiac remodeling and dysfunction. Anderson *et al* (123) reported that treating 24-month-old mice with the senolytic navitoclax for 1 month suppressed cardiomyocyte senescence, reduced hypertrophy, fibrosis and LV mass. Similarly, Dookun *et al* (124) demonstrated that ischemic reperfusion triggers senescence, and administering navitoclax 4 days after injury improved vascularization and functional recovery. Although effective in eliminating senescent cells, the hematological adverse effects of navitoclax, such as thrombocytopenia, limit its use with other therapies (125).

Lee *et al* (126) demonstrated for the first time that the local delivery of the senolytic drug, ABT263, using poly lactic-co-glycolic acid-derived nanoparticles can clear stress-induced senescent cells following myocardial ischemic reperfusion injury. The findings included reduced cardiac remodeling and recovery of cardiac function (126). Although human trials regarding senolytics remain limited, preclinical experimental studies have demonstrated the safe administration and potential cardioprotective effects of senolytic therapy (126).

Another strategy explores gene therapy to restore myocardial cellular function. The SERCA-LVAD trial confirmed that direct delivery of gene therapy is safe and feasible in patients with LVAD. The gene was effectively taken up by the myocardium. However, despite the improvement in biological markers, patients did not achieve full recovery, likely due to persistent structural remodeling, timing of intervention, and incomplete cardiomyocyte uptake (10).

## 6. Delivery devices, imaging and integration with surgical practice

### Delivery devices and safety considerations

Therapeutics can be delivered to the myocardium using three primary techniques: Intramyocardial/intracoronary catheters, epicardial surgical platforms and percutaneous or robotic systems. Each approach has its own advantages in terms of procedure, technical limitations and safety concerns.

*Delivery devices*. Intramyocardial and transendocardial catheters enable the direct administration of therapeutic drugs into the myocardial tissue via direct myocardial puncture or percutaneous endocardial access. These technologies improve regional bioavailability and retention by facilitating localized delivery (20).

Intracoronary infusion platforms include selective intracoronary and balloon occlusive catheters, which deliver therapeutics to the coronary circulation, achieving better myocardial distribution without direct tissue penetration (108).

Epicardial deployment systems utilize procedures such as CABG for the prompt delivery of micrograft sheets placed on an ECM and secured on the cardiac surface. These allow precise positioning and integration of extracellular matrix scaffolds, often with improved local paracrine support (127).

Percutaneous scaffold/patch delivery systems comprising minimally invasive epicardial or percutaneous placement of scaffold and hydrogel platforms offer potential reductions in surgical trauma (128).

Hybrid robotic or thoracoscopic applicators combine precision and stability, potentially reducing operator-dependent variability and enabling precise insertion in confined cardiac spaces (129).

Cardiothoracic surgery (CTS) teams employ surgical routes, such as CABG to implant micrograft patches (127). Hybrid CTS/interventional cardiology strategies have also emerged, combining surgical access with catheter-based delivery to increase precision and therapeutic reach (92).

*Safety considerations*. All myocardial delivery approaches carry certain risks. Myocardial injections and catheter-based infusions have potential risks for arrhythmias, embolization, myocardial perforation and subsequent tamponade (20), whereas implanted patches and grafts carry a risk of device material incompatibility and prolonged immunosuppression (127). Open surgical procedures pose the risk of wound infection due to barriers in maintaining sterility. Vector-based or cell therapies may trigger immunogenic reactions, requiring diligent patient monitoring and material selection (130). Therapeutic delivery to the myocardium can be categorized by delivery route and procedural context, with each platform presenting distinct advantages and safety limitations as presented in Table IV.

### Imaging and intraoperative guidance. Pre-operative planning

Pre-operative imaging is of utmost importance in forming an effective surgical strategy and device placement. Pre-operative computed tomography (CT) and CT angiography with or without contrast can be utilized to visualize accurate imaging of coronary structures (131). Xu *et al* (132) observed that combined usage of delayed-enhancement cardiac MRI or LGE with fluorine-18 fluorodeoxyglucose positron emission tomography enhanced accuracy in detecting viable myocardium prior to a surgical procedure. Similarly, LGE MRI, along with T1 and T2 mapping, reveals myocardial tissue status and diffuse changes (133). Overall, these imaging modalities allow risk assessment, tailored surgical approaches and improve pre-operative evaluation.

*Intraoperative guidance*. Real-time imaging intraoperatively enables improved precision. Transesophageal echocardiography (TEE) and 3D TEE remain indispensable for imaging, intraoperative guidance, and evaluation of complications (134). Fritz *et al* (135) reported a case study regarding ultra-high frequency epicardial ultrasound during CABG for apt visualization of coronary anatomy and graft placement. Preclinical and clinical studies have demonstrated the efficacy of electromechanical mapping using NOGA catheters in locating viable myocardial injecting sites (136). Fluorescent cardiac imaging systems provide high-resolution intraoperative angiography, thus allowing the real-time assessment of coronary vessels, graft patency and the myocardial perfusion status (137).

*Post-operative imaging*. Imaging has been performed at <24, 24-72 and >72 h post-surgically using transthoracic echocardiography/TEE. This proved to be an excellent diagnostic modality for detecting post-operative cardiac tamponade when performed after 24 h of surgery (138). Hwang *et al* (4) suggested using cardiac MRI along with LGE for intermediate follow-up at 3 months and long-term follow-up at 12 months, respectively. This imaging modality allows meticulous inspection of myocardial wall mobility and recovery of function (4). Multimodal evaluation assists in determining procedural success and myocardial viability over time.

### Hybrid operating room workflows and peri-operative protocols. Key peri-operative considerations

Peri-operative management necessitates anti-coagulation protocols with intraoperative heparinization and activated clotting time targets aligned to EACTS/EACTA/EBCP guidelines (139). Infection control requires the early administration of antibiotics, such as cefazolin within 60 min prior to incision and vancomycin for the methicillin-resistant *Staphylococcus aureus*-associated risk (140). In addition to antibiotic prophylaxis, the initiation and continuation of immunosuppression are mandatory when allogenic cells are delivered (141). The StimAOD multicenter research supports organized direct oral anticoagulant cessation and recovery protocols, indicating no increase in thromboembolic or hematoma risk as compared to more aggressive bridging techniques (142).

Pandozi *et al* (143) emphasized the standardized treatment of drains, chest tubes and early intensive care unit monitoring to detect hemodynamic or rhythm anomalies, in compliance with recognized enhanced recovery after cardiac surgery-style pathways. In addition, the SMARTEL trial demonstrates improved post-operative adverse event detection using continuous telemetry operations (144).

*Recommended imaging and follow-up schedule*. Graft safety, changes in ventricular structure, and functional recovery following surgery are assessed using baseline cardiac MRI; follow-up CMR tests are performed at 3 and 6 months (62,127). Other evidence adheres to a multimodal approach, using baseline cardiac CT, fluorodeoxyglucose-positron emission tomography and echocardiography with follow-up imaging at 6 and 12 months to assess structural changes in myocardial tissue following regenerative therapies (145). Biomarker monitoring, troponin, BNP/NT-proBNP and C-reactive protein (CRP) are used along with imaging to identify myocardial inflammation (146).

*Regeneration-specific workflow and hybrid operating room integration*. Incorporating regenerative therapies into surgical workflows requires precise intraoperative protocols. CABG procedures combined with epicardial biologic application using GMP-compliant patches, spray systems, and cell-seeded scaffolds. Nummi *et al* (127) reported on on-table processing of autologous atrial biopsies, which were minced, homogenized and applied onto an ECM sheet under strict sterility before epicardial placement. Prat-Vidal *et al* (62) highlighted the PeriCord allogeneic pericardial matrix, produced under full GMP policies, transported in a cryopreserved state and thawed in the operating room with guaranteed sterility and viability protocols prior to sutured implantation. Adipose-derived cell and scaffold approaches were demonstrated by Kędziora *et al* (130), emphasizing hybrid workflows that incorporate revascularization and regenerative enhancement. Hybrid operating rooms require sterile thawing and mixing areas, strict adherence to sterility protocols and peri-operative immunomodulation strategies (130). Steady timing (post-bypass, pre-closure), patch fixation, and intraoperative imaging aid safe and reproducible biologic delivery.

## 7. Safety, ethical, regulatory and reoperation considerations

### Safety signals observed in trials

To evaluate the safety and effectiveness of heart regenerative therapies, numerous clinical trials, including stem-cell-based therapies in MI and ischemic cardiomyopathy, have been carried out. Short- to mid-term results generally demonstrate an overall acceptable safety profile for MSC and adult stem-cell therapies when compared to control groups, with no notable increase in significant adverse cardiac events, stroke, reinfarction, or cardiovascular death (147-150). Multiple systematic reviews and meta-analyses have demonstrated moderate improvements in LV ejection fraction, although infarct size reduction was absent or minimal in several studies (147,149,150). This implies that rather than actual myocardial regeneration, notable functional improvements may be due to paracrine or immunomodulatory effects (147,149,150). The relatively low frequency of tumor formation and malignant transformation in published trials supports short-term oncologic safety; nevertheless, long-term surveillance data are still limited, and delayed adverse effects cannot be ruled out (149,150,151). Despite early warning indicators, unresolved long-term concerns persist, particularly those linked to immunogenicity, genetic instability and arrhythmogenic capability. Experimental and translational research indicates that electrical heterogeneity at host-graft interactions and electrophysiological immaturity of transplanted cardiomyocytes could contribute to ventricular arrhythmias, even if this risk has not yet been consistently observed in clinical trials (151,152). The possible risks of uncontrolled differentiation and immunological reactions to allogeneic cells or viral vectors further emphasize the need for extended monitoring (151,152). Therefore, current trial frameworks favor rigorous pre-procedural arrhythmia risk assessment, immunological screening, and systematic safety monitoring before a comprehensive catalog of rare adverse events (151,152).

### Reoperation and long-term durability

Long-term durability is a crucial, yet unexplored component of cardiac regeneration treatment, since a considerable proportion of patients with ischemic cardiomyopathy eventually require recurrent heart surgery. Although short- and mid-term trials of stem-cell-based therapies have acceptable safety profiles, the long-term behavior of biomaterial patches, hydrogels, and cell-seeded scaffolds is still unclear (147-150). As many regenerative medicine studies have relatively short follow-up periods, structured long-term surveillance frameworks, including specialized registries, have been proposed to document reoperation rates, graft persistence, device-related complications and late adverse events throughout the patient lifespan (153-155). From a surgical perspective, the application of regenerative constructs to the epicardial surface may create regions where the native myocardium and implanted materials are structurally mismatched, promote adhesions, or alter the architecture of scars, rendering subsequent surgeries more challenging. Due to the partial degradation or persistence of biomaterial residues, anatomical planes may be narrowed, pericardial dissection may be distorted, and repeat sternotomy may become more difficult, problems that are typically overlooked in early-phase effectiveness trials (156,157). Additionally, fibrosis, inflammatory responses, or chronic mechanical fatigue may compromise long-term therapeutic effect (157).

### Regulatory and ethical landscape

The regulatory supervision of cardiac regeneration therapies is substantially more complex than that of conventional pharmacological agents due to biological variability, manufacturing heterogeneity and long-term uncertainty in stem-cell-based research (147,149-151). In the USA, the FDA regulates cell and gene therapies under the Biological Products framework. Rapid access is made possible by the Regenerative Medicine Advanced Therapy designation, which is reliant upon robust Chemistry, Manufacturing and Controls data, tumorigenicity testing, and a 15-year post-marketing surveillance period for gene-modified products (158,159). Combination products, including biomaterial scaffolds and cell-seeded patches, are subject to coordinated biologic-device evaluation. The European Medicines Agency classifies these therapies as Advanced Therapy Medicinal Products in Europe under Regulation no. 1394/2007, which calls for centralized approval, traceability, and extensive pharmacovigilance (160,161). As regards cost, fair access, long-term safety obligations and informed permission for experimental interventions, regenerative therapies pose particular ethical challenges. Ethics committees and institutional biosafety committees are crucial for ensuring that patients are properly informed about the experimental status, uncertain durability, and potential late consequences of a number of regenerative designs (152-155). Mandatory registry participation, data transparency and longer follow-up periods are increasingly viewed as ethical imperatives due to inadequate reporting of late adverse events in short-duration studies (153-155).

## 8. Key barriers to translation and research priorities

### Key barriers to translation. Biological barriers

Despite decades of progress, poor transplant cell engraftment and survival remain the primary biological barriers to cardiac regeneration. In the post-MI setting, inflammation, ischemia, mechanical strain and washout pressures cause most transplanted cells to be quickly lost, regardless of the cell source (162,163). Long-term retention gains are still very minor, despite the investigation of fibrin gels, injectable hydrogels, and synthetic or natural scaffolds as supplementary delivery techniques (162,164). Liew *et al* (162). stated that persistent inflammation and mechanical stress significantly restricted engraftment in the physiologically hostile zone of the infarcted myocardium. Delivery-route-specific restrictions, such as cell leakage after intramyocardial injection and off-target sequestration after intravenous administration, complicate this problem (163). Additionally, the intrinsic capacity of the adult human heart to integrate exogenous cells is restricted (165).

Chronic cardiomyopathy raises further challenges as ECM remodeling and fibrosis impede chemotactic signaling and cellular anchorage (163). Although early post-MI chemokine gradients may temporarily increase homing, reperfusion damage and signal fading hinder long-term survival (163). There is still a substantial unsolved safety-efficacy trade-off, since cardiomyocytes produced from embryonic or induced pluripotent stem cells have been frequently associated with ventricular arrhythmias related to engraftment (163,166). Comparisons between cell-derived secretomes and cell-based therapies indicate biological activity, but they also highlight persistent uncertainty regarding the main therapeutic mechanism, for instance, engraftment, immunomodulation, paracrine signaling, or indirect remodeling effects (167).

New strategies focus on preconditioning cells, generating pro-survival matrices, enhancing cardiomyocyte electromechanical development and prioritizing acellular or hybrid techniques where needed to reduce the risk of arrhythmia (164,166,167).

*Manufacturing and standardization hurdles*. The production of regenerative medicines in accordance with GMP standards poses significant translational challenges. It remains difficult and resource-intensive to guarantee sterility, batch-to-batch repeatability, and uniform biomaterial composition or cell loading (164,168). Complex quality-control methods and limited scalability impede widespread clinical use (168). Reproducibility is further hampered by raw-material variability, which includes donor-dependent differences in cell phenotype, matrix polymers and hydrogel composition. This results in varied treatment outcomes across studies (164). Moreover, poor reprogramming efficiency causes additional translational difficulties, and retroviral or integrated transgene delivery methods create safety concerns (165). Successful scale-up requires standardized release criteria, non-integrating gene-delivery technologies, modular bioprocessing pipelines, and early manufacturability inclusion into trial design (164,168).

*Endpoint heterogeneity*. Surrogate imaging markers such as LV volumes, EF, extracellular volume and myocardial strain are widely used, yet many lack formal clinical qualification as regulatory endpoints (169). Underlying comorbidities, core-lab adjudication practices, and site-specific imaging protocols further increase variability (169,170). Systematic analyses of trial registries reveal marked heterogeneity, with some studies reporting multiple primary endpoints while others rely on single surrogates; composite endpoints further complicate cross-trial comparisons (170). Inconsistencies in imaging modalities, including MRI, echocardiography, CT and functional exercise testing, and wide variation in follow-up timing windows undermine statistical power and regulatory interpretability (170). Pre-specification of standardized, regulator-endorsed imaging and clinical endpoints, along with harmonized acquisition protocols and fixed follow-up windows, is essential to enable comparison and meta-analysis (169,170).

*Trial design barriers*. Non-randomized or underpowered early phase trial design remains common, and varied inclusion criteria lead to patient substrates being mixed (171). These variations span critical biological dimensions such as ischemic timing, MI type, baseline LV size, or EF, producing heterogeneous patient substrates (171). Some trial design considerations, such as the documentation of intraoperative processes, introduce procedural variability (127). This variability arises from key operative details, such as injection depth, cell dosing volume, or orientation of patches. Small deviations in these parameters can significantly alter cell retention/distribution and efficacy (171). The lack of clear reasoning for sample size or phase transitions complicates design choices for multicenter trials (170).

*Regulatory complexity*. Early engagement with regulatory bodies such as the FDA or European Medicines Agency (EMA) is mandated due to historic poor endpoints and insufficient data found in earlier trials. Regulators require early knowledge on the target population, safety protocols, potency assays, and trial advancement criteria (172). The lack of standardized potency assays for cardiac biologics applies pressure to define potency for properties that are not usually measurable. In addition, long-term follow-ups have caused a time and financial burden (172). Regulators also usually request data regarding immunotoxicity, tumorigenicity and biodistribution prior to first-in-human trials, followed by post-market surveillance obligations (173).

*Funding and industry*. Private-sector investment is further restricted by high GMP costs, complex quality-control standards, and uncertainties over scalability and reimbursement (43). It is difficult for promising treatments to attract industry partners if manufacturability and economic feasibility are not considered at an early stage. Early translational efforts must include scalable production, clear commercialization paths and cost-effectiveness in order to sustain industry engagement (43).

### Critical research priorities for surgical translational teams. Standardized outcome endpoints (imaging and clinical)

The ReGenHeart Phase II trial illustrates the necessity for uniform clinical and imaging endpoints. Trials should specify metrics, time frames and the use of the same imaging criteria in advance to ensure comparability (174). Uniform MRI protocols are particularly crucial as non-invasive imaging is essential for evaluating regenerative therapies. In a previous study, molecular MRI with collagen- and elastin-specific contrast agents was used to monitor fibrosis following chordin-like 1 therapy, demonstrating how targeted imaging can improve mechanistic understanding (175). Additional precision measurements, such as LV circumferential strain and quantification of histologic fibrosis (e.g., Masson's trichrome in preclinical investigations), can direct biomaterial optimization (176). NYHA class, heart failure hospitalization, CRP-stratified functional status and standardized 6-minute walk distance protocols are examples of clinical outcomes that should be regularly included. In addition to supporting regulatory qualification, these measures record patient-centered changes (31,177).

*Imaging protocols and timing frameworks*. Trials should have uniform imaging windows to capture early remodeling, stabilization and long-term effects. Large animal models demonstrate the feasibility of 3- and 6-month CMR follow-ups, while this is not true for human trials (68).

*Multi-center translational networks*. The POSEIDON trial provides the optimal example of the advantages of inter-institutional cooperation (178). Shared recruitment frameworks, unified production pipelines, and uniform delivery methods improve trial integrity and reduce site-specific variability. Similar networks will be required for future biologic-device hybrid trials, where core-lab imaging, bioprocessing, and surgical delivery must all operate seamlessly (178).

### Reasons why early-stage promising trials failed in the later phases

Several recurring, interrelated factors explain why encouraging early-phase results often did not translate into later-stage clinical success: i) Delivery and retention limitations: A number of preclinical models overestimate myocardial retention and underestimate washout; thus, biologics that appear effective in small animals fail to achieve therapeutic exposure in human myocardium (21,23,32). ii) Model and species differences: Rodent and small-animal physiology, immune responses and infarct biology differ substantially from human disease, producing inflated efficacy signals that do not scale to patients (33,34). iii) Endpoint and trial design issues: Early trials frequently relied on surrogate imaging endpoints, small sample sizes, heterogeneous inclusion criteria and variable imaging protocols, reducing statistical power and regulatory confidence (169,170). iv) Safety and mechanistic uncertainty: Arrhythmogenicity, immune reactions and unclear mechanisms (paracrine vs. remuscularization) created safety concerns and ambiguous dose-response associations that complicated phase advancement (163,166,167). v) Manufacturing and scalability constraints: The lack of standardized potency assays, batch variability and high GMP costs impeded consistent product quality and commercial feasibility (43,164,168). vi) Regulatory and economic misalignment, uncertain reimbursement pathways and long post-market surveillance burdens reduced industry willingness to invest in large definitive trials (43,172). Together, these factors demonstrated that biological promise alone is insufficient: Robust delivery platforms, harmonized endpoints, standardized potency metrics and early manufacturability planning are essential to convert early signals into reproducible, late-phase success.

### Practical solutions to accelerate translation

Early regulator engagement and endpoint harmonization are required. Holding pre-IND/IMPD meetings is essential to agree target populations, safety monitoring and acceptable imaging/clinical endpoints; pursuing formal endpoint qualification may also be helpful where feasible (169,170,172,173). It is also essential to design for manufacturability. This requires GMP-compatible processes, non-integrating technologies and predefined release criteria during preclinical planning to reduce scale-up risk (164,168).

*i) Modular, centralized manufacturing*. The use of automated, closed-system bioprocessing and regional GMP hubs or consortium models may improve batch consistency and lower per-unit costs (164,168,178).

*ii) Adaptive, platform trial designs and standardized procedures*. It is necessary to adopt master-protocols with shared controls, core-lab imaging and published procedural checklists (injection depth, patch orientation and dosing) to reduce variability and accelerate comparisons (127,170,171,174).

*iii) Early economic planning and de-risking*. Health-economic modelling should be performed at an early stage, public-private partnerships or milestone funding should be pursued and registry-based post-market evidence is necessary to support reimbursement (43,172).

## 9. Future directions

The future of heart regenerative medicine is moving toward an integrated bio-surgical approach that integrates cellular, molecular, and surgical breakthroughs to promote structural restoration rather than symptomatic treatment (179). MSC-derived exosomes employed in CABG have improved mitochondrial function, reduced fibrosis, and controlled inflammation in preclinical models (180,181). Next-generation cardiac patches combine energy storage, electrical stimulation, and diagnostic signal monitoring to enhance electroactivity and preserve ventricular shape in animal models (59,61). Hydrogel-based and bioadhesive epicardial patches improve intraoperative practicality and enable targeted delivery of cells, RNA, or cytokines (182). Emerging technologies, such as AI-guided imaging optimize patch placement, risk stratification, and outcome prediction, whereas modRNA therapies aim to promote cardiomyocyte proliferation and enhance heart repair (183-185).

Standardized large-animal models remain helpful in evaluating immunogenicity, arrhythmogenic potential and engraftment (186). Preclinical techniques that mimic human perfusion and surgical settings are crucial for improving translational integrity. First-in-human research, such as synthetic heart muscle allografts, indicates the importance of tumorigenicity testing, perfusion MRI and arrhythmia surveillance to meet regulatory requirements (79,187). Ethics and regulations remain crucial. Informed consent should explicitly state that treatments are experimental and carry hazards such as electrical mismatch, tumorigenicity, or arrhythmias (158,188,189). Teratoma formation is a concern associated with iPSC-derived treatments, but embryonic stem cell-based approaches raise concerns related to genomic instability (188,189). Unresolved challenges include genetic tampering, donor permission, embryo destruction, and long-term monitoring responsibilities (158,190). Registries and post-trial care are essential for collecting data and patient safety (191).

Scalability and cost remain challenges. Compared to autologous iPSC lines, which are expensive and take time to develop, allogeneic ‘off-the-shelf’ products provide faster, more standardized, and potentially more accessible possibilities (192). In order to enhance patient selection, procedural planning, and post-intervention monitoring, translational workflows are simultaneously increasingly integrating AI-enabled cardiovascular imaging and data-driven diagnostic workflows. This may improve the use of regenerative treatments in clinical settings (193,194). In conclusion, the integration of molecular therapies, tailored biomaterials, enhanced imaging, and innovative surgery is advancing heart regenerative medicine. Achieving clinical translation requires standardized preclinical-to-clinical procedures, ethical and regulatory clarity, and strategies that ensure these therapies are widely accessible.

## 10. Conclusion

In conclusion, in preclinical models of myocardial injury, a variety of molecular and regenerative techniques have shown the capacity to improve heart function. Even while early clinical trials of cell treatments and growth factors have demonstrated some safety and benefit, full cardiac muscle repair remains unachievable. Exosome-based therapeutics, synthetic cardiac patches, gene transfer of cardioprotective proteins, and cardiomyocyte grafts produced from pluripotent stem cells are just a few of the cutting-edge techniques being evaluated in a number of clinical trials. The translational landscape is rapidly evolving. Research is also looking into synergistic combinations, including combining cell transplantation with specific molecular therapies to overcome individual limitations. Recent early-phase human studies discussed above, including synthetic cardiac tissues and next-generation gene therapies, reflect this progress. Although challenges remain (such as limited cell engraftment, immunological reactions and the possibility of arrhythmia), there is reason for anticipation owing to the rapid growth of technology and growing understanding of heart physiology. In the coming years, integrated strategies guided by molecular diagnostics and improved imaging may enable truly regenerative therapeutics. Coordinated translational efforts will determine the safety and efficacy of delivering these improvements to patients, which could transform the management of MI and HF.

## Figures and Tables

**Table I:** Major clinical trials evaluating regenerative therapies post-MI and ischemic cardiomyopathy.

Trial name	Therapy/cell type	Delivery route	Sample size/population	Key outcomes	Limitations	(Refs.)
REPAIR-AMI	Autologous BM-MNCs	Intracoronary infusion	Not specified	EF improvement 3-4% with clinical benefits beyond EF changes	Heterogeneity, reliance on echocardiography, modest efficacy	(16,21)
BM-MNC Meta-analysis (22 RCTs)	Autologous BM-MNCs	Intracoronary infusion	n=956 AMI patients (pooled)	LVEF +2.10% (95% CI 0.68-3.52), LVESV -4.05 ml, infarct size -2.5%; MRI-derived outcomes (n=9) showed no improvement; No impact on MACCE at 6 months	Heterogeneity in protocols, reliance on echocardiography, modest efficacy	(21)
BAMI phase III	Autologous BM-MNCs	Intracoronary infusion	n=375 (LVEF ≤45%)	2-year mortality 3.26% vs. 3.82% (placebo); HF hospitalization 2.7% vs. 8.1% (placebo)	Insufficient to detect clear efficacy conclusions	(22)
MSC meta-analysis (13 RCTs)	Autologous MSCs	Intracoronary, intramyocardial, or intravenous	n=956 AMI patients	LVEF enhancement +3.78% (95% CI 2.14-5.42); early treatment (within 1 week post-AMI) +5.7%; no significant effects on LV volumes or HF rehospitalization	Heterogeneity in cell doses (2.3-85×10⁶), infusion timing, follow-up (6-182.6 months)	(23)
POSEIDON	Allogeneic and autologous MSCs	Transendocardial injection	Not specified	Scar reduction, safety, functional improvements (particularly transendocardial delivery)	Moderate benefits influenced by delivery-related variability	(25)
TAC-HFT	Allogeneic and autologous MSCs	Not specified (chronic ICM)	Not specified	Scar reduction, safety, functional improvements	Moderate benefits influenced by delivery-related variability	(25)
MSC-HF	MSCs	Not specified	Not specified	Better symptoms but no notable EF effect	Limited by delivery-related variability	(25)
SCIPIO	Autologous c-kit^+^ CPCs	Intracoronary injection	Ischemic cardiomyopathy patients	LVEF improved from 30% to roughly 38%; reduced infarct size	Limited preclinical-to-clinical translation (mostly preclinical data); large knowledge gap	(26)
CADUCEUS	Cardiosphere-derived cells (CDCs)	Not specified (chronic ICM)	Not specified	Very minor EF alterations; significant scar reduction (-12 g); increased viable myocardium	Limited efficacy on EF; large knowledge gap vs. BM-MNC/MSC	(27)
ALCADIA	CDCs + gelatin hydrogel	Intramyocardial/patch delivery	Limited sample size	Improved EF and symptomatic improvement	Small sample size; limited clinical translation data	(28)
PROMETHEUS	Autologous MSCs	Intramyocardial injection during CABG	Feasibility study	Enhanced regional wall motion and procedural safety	Not powered for efficacy; feasibility study only	(29)
CONCERT-HF	CDCs and MSCs	Intraoperative (CABG setting)	Not specified	Improvements in quality-of-life outcomes, reduction in HF hospitalization; marginal improvements in ventricular function	Only marginal functional improvements	(30)
DREAM-HF (Phase III)	CDCs and/or MSCs	Intraoperative (CABG setting)	Large phase III cohort	Reductions in inflammatory biomarkers, fewer HF hospitalizations (particularly in high inflammatory status); NO improvement in primary composite outcome	No improvement in primary composite outcome; primarily paracrine/immunomodulatory effects	(31)
TMR + BMSC (FDA BB-IND 14758)	Autologous bone marrow MSCs (BMSCs)	Intramyocardial injection during CABG	Not specified	Feasible and short-term safety demonstrated; increased QOL, reduced angina	Short-term follow-up only; limited efficacy data	(12)
CL2020 (muse cell therapy)	Allogeneic muse cells	Intracoronary and intravenous delivery	STEMI patients	Well tolerated; functional improvement in STEMI patients	Early feasibility data; limited long-term follow-up	(8,9)
Umbilical cord-derived MSC trial	Allogeneic umbilical cord-derived MSCs	Intracoronary and intravenous delivery	STEMI patients	Improved cardiac function at the 1-year follow-up	Limited efficacy on remuscularization; likely paracrine mechanism	(8,9)

REPAIR-AMI, reinfusion of enriched progenitor cells and infarct remodeling in acute myocardial infarction; BM-MNCs, bone marrow-derived mononuclear cells; RCTs, randomized controlled trials; EF, ejection fraction; LVEF, left ventricular ejection fraction; LVESV, left ventricular end-systolic volume; MACCE, major adverse cardiac and cerebrovascular events; MSCs, mesenchymal stem cells; HF, heart failure; SCIPIO, stem cell infusion in patients with ischemic cardiomyopathy; CPCs, cardiac progenitor cells; CABG, coronary artery bypass grafting; STEMI, ST-elevation myocardial infarction.

**Table II:** Gene and RNA therapy platforms for cardiac repair with clinical/preclinical evidence.

Therapy/platform	Target/pathway	Supporting trial/study	Outcomes/effects reported	Limitations/failure points	(Refs.)
AAV1-SERCA2a (Calcium ATPase)	SERCA2a upregulation in HFrEF; restore calcium handling	CUPID Phase 1/2 (n=9, intracoronary): favorable safety, improved functional class, NT-proBNP reduced, LVESV reduced.	Acceptable safety profile; early biological activity; investigator-reported benefits	Limited cardiotropism; dose ceiling (intracoronary limitation); inadequate empty capsids (reduced immune decoy function); immunogenicity; CUPID-2 Phase 2b (n=250) failed: neutral clinical outcomes, no HF event reduction despite safety	(87-89)
AAV2i8-based AB-1002	Enhanced myocardial transduction vs. AAV1; cardiotropic vector	Phase I trial (n=11 HF patients, systemic delivery)	LVEF +4-7% at 6 months; NT-proBNP reduced; 6-minute walk test +28-40 m average; better myocardial uptake than AAV1	Early-phase small sample; long-term efficacy unknown; immunogenicity monitoring ongoing; scalability concerns	(91,92)
AAV9-based MyoAAV 4A and 4E	Improved cardiotropism and transduction efficiency over AAV1	Preclinical/translational studies (human myocardium *ex vivo*)	Higher transduction efficiency with reduced off-target uptake vs. AAV1	Preclinical stage; immunogenicity concerns; long-term integration risk	(91)
SERCA-LVAD (AAV-SERCA2a in LVAD patients)	SERCA2a delivery in mechanically supported hearts	Clinical trial: Safe and feasible gene delivery in LVAD patients	Favorable molecular and cellular alterations; safety established; effective myocardial uptake	No full functional recovery achieved; incomplete cardiomyocyte uptake; persistent structural remodeling; timing of intervention suboptimal	(10)
LNP-mRNA (ionizable lipid nanoparticles with mRNA)	Vasculogenic and cytoprotective factors; VEGF-mRNA; modified mRNA encoding reparative proteins	Anttila *et al* (n=11, intramyocardial VEGF-mRNA during CABG)	Enhanced perfusion, decreased fibrosis, improved capillary density, neovascularization, improved cardiomyocyte survival (preclinical)	Transient expression; manufacturing scalability; lipid toxicity concerns; preclinical-dominant evidence	(83,86,100)
siRNA + ASOs (small interfering RNA + antisense oligonucleotides)	Post-transcriptional silencing: RAGE-siRNA, MIAT-siRNA, Nucleophosmin 1 ASO; inclisiran (PCSK9), volanesorsen (ApoC-III)	Preclinical (cardiac targeting peptide-EV + RAGE-siRNA, MIAT-siRNA); Phase 3 trials (inclisiran, volanesorsen in cardiometabolic disease)	Reduced inflammatory infiltrates and fibrosis (RAGE); reduced apoptosis, improved post-MI function (MIAT); improved cardiomyocyte repair capacity (NPM1 ASO); promising cardiometabolic outcomes (inclisiran, volanesorsen)	Preclinical stage for MI-specific siRNA; limited direct cardiac translation; delivery optimization needed; off-target effects possible	(93-96,99)
miR-93 delivery (microRNA modification)	Hippo-Yes-associated protein (YAP) pathway activation	Preclinical models	Increased angiogenesis, reduced infarct size and fibrosis	Preclinical stage; delivery platform optimization needed; immunogenicity of miR carriers unknown	(101)
CRISPR-Cas13d (hpCas13D)	Allele-specific cleavage of myosin heavy polypeptide 7 (genetic correction)	Patient-derived cardiomyocytes (ex vivo)	Restored contractility in diseased cardiomyocytes	Early-stage gene editing; specificity concerns (Cas9 showed non-specific uptake); off-target effects; *in vivo* delivery challenges; safety monitoring critical	(102,104)
Direct intramyocardial gene injection (VEGF-mRNA, other mRNAs)	VEGF-mRNA enhancing myocardial perfusion and remodeling	Anttila *et al*, clinical trial (n=11, intramyocardial injection during CABG)	Enhanced perfusion, decreased fibrosis, improved capillary density	Localized delivery only; limited systemic reach; variable tissue uptake; efficacy dependent on injection technique	(86)
Epicardial geneeluting patches and bioengineered scaffolds	Sustained localized release of gene/mRNA vectors	Preclinical; integration with CABG workflows	Improved spatial control; extended therapeutic duration; reduced systemic toxicity	Manufacturing complexity; standardization challenges; durability questions; clinical translation limited	(43)

AAV, adeno-associated virus; SERCA2a, sarcoplasmic/endoplasmic reticulum calcium ATPase 2a; HfrEF, hear failure with reduced ejection fraction; LVESV, left ventricular end-systolic volume; LVEF, left ventricular ejection fraction; HF, heart failure; LVAD, left ventricular assist device; LNP, lipid nanoparticle; CABG, coronary artery bypass grafting.

**Table III:** Comparative overview of regenerative and molecular therapies for myocardial repair.

Therapy	Primary mechanism	Typical efficacy (ΔLVEF/scar)	Major safety concerns	Clinical readiness (phase)	(Refs.)
BM-MNCs (bone marrow mononuclear cells)	Paracrine immunomodulation; angiogenesis.	LVEF= +2.1% (meta-analysis); REPAIR-AMI +3-4%	Procedural risks; modest efficacy; heterogeneity in outcomes.	Phase II-III; BAMI inconclusive.	(16,21,22)
MSCs (mesenchymal stromal cells)	Paracrine/exosome release; promote angiogenesis and anti-apoptosis.	LVEF= +3.78% (meta-analysis); early treatment larger benefit.	Variable retention; limited remuscularization; immune/allogeneic issues	Phase II trials; mixed Phase III signals.	(23,25)
CPCs/CDCs (cardiac progenitor/cardiosphere cells)	Stimulate viable myocardium via paracrine and niche effects.	Scar reduction reported (e.g., CADUCEUS-12 g); EF improvement reported in SCIPIO.	Small sample sizes; uncertain durability.	Early clinical (feasibility/small RCTs)	(26,27)
iPSC-CMs and engineered cardiac patches	Direct remuscularization; electromechanical integration.	Preclinical large-animal LVEF improvements; early human safety signals.	Arrhythmogenic risk; immune rejection; scalability and durability concerns.	Preclinical → first-in-human patches; early feasibility studies.	(33-37,62)
Exosomes/EVs (cell-free vesicles)	Deliver bioactive cargo (miRNA, proteins) for angiogenesis, anti-fibrosis.	Preclinical: improved angiogenesis and LV function; clinical MI data limited.	Low tissue retention; manufacturing yield and targeting challenges.	Preclinical → early translational studies; some non-MI clinical trials.	(14,17,19,38,40)
Gene and RNA therapies (AAV, siRNA, ASO, mRNA)	Molecular modulation of Ca²^+^ handling, inflammation, fibrosis, metabolism.	Mixed: SERCA2a trials inconsistent; siRNA/ASO show target engagement and preclinical benefit.	Vector tropism/off-target uptake; immune responses; delivery efficiency.	Several clinical trials (early phases); some modalities in advanced cardiometabolic trials.	(10,87,88,90-96,99)
Biomaterials/adhesive patches (hydrogels, ECM, conductive scaffolds)	Mechanical support; local paracrine retention; improved electrical coupling.	Case reports/first-in-human show functional improvement (anecdotal); preclinical scar reduction.	Thrombogenicity, infection, reoperative adhesions; long-term durability unknown.	Early clinical implants and feasibility studies; ongoing trials.	(59,62-64,72,73)

AAV, adeno-associated virus; ASO, antisense oligonucleotide; BM-MNCs, bone marrow mononuclear cells; CADUCEUS, Cardiosphere-derived autologous stem cells to reverse ventricular dysfunction; CDCs, cardiosphere-derived cells; CPCs, cardiac progenitor cells; ECM, extracellular matrix; EF, ejection fraction; EVs, extracellular vesicles; iPSC-CMs, induced pluripotent stem cell-derived cardiomyocytes; LV, left ventricular; LVEF, left ventricular ejection fraction; MI, myocardial infarction; MSCs, mesenchymal stromal cells; mRNA, messenger RNA; REPAIR-AMI, Reinfusion of Enriched Progenitor Cells and Infarct Remodeling in Acute Myocardial Infarction; RNA, ribonucleic acid; RCTs, randomized controlled trials; SCIPIO, stem cell infusion in patients with ischemic cardiomyopathy; SERCA2a, sarcoplasmic/endoplasmic reticulum calcium ATPase 2a; siRNA, small interfering RNA.

**Table IV:** Myocardial therapeutic delivery platforms: Routes, advantages and safety considerations.

Device/platform	Delivery route	Main benefits	Key risks/limitations	(Refs.)
Intramyocardial/transendocardial catheters	Percutaneous endocardial or direct myocardial injection	Localized therapeutic delivery, improved retention	Arrhythmias, embolization, myocardial perforation, tamponade	(20)
Intracoronary infusion platforms	Coronary artery infusion (selective or balloon-occlusive)	Broad myocardial distribution without tissue puncture	Coronary embolization, local ischemia, limited penetration into infarcted tissue	(108)
Epicardial deployment systems (surgical)	Open-heart access (CABG, median sternotomy)	Precise patch placement, integration with ECM scaffolds, enhanced local paracrine effects	Surgical trauma, infection, immunogenicity, prolonged recovery	(127)
Minimally invasive scaffold/hydrogel delivery	Percutaneous or thoracoscopic epicardial placement	Reduced surgical trauma, localized patch delivery	Limited visual guidance, anchoring challenges, risk of incomplete placement	(128)
Hybrid robotic/thoracoscopic applicators	Robotic-assisted or uniportal VATS	High precision, operator-independent placement, reduced invasiveness	Learning curve, device complexity, procedural cost	(129)

## Data Availability

Not applicable.
